# Predictors of In-Hospital Mortality for Stroke in Douala, Cameroon

**DOI:** 10.1155/2014/681209

**Published:** 2014-02-25

**Authors:** N. Y. Mapoure, C. B. Tchaleu Nguenkam, H. B. Mbatchou Ngahane, A. Dzudie, A. Coulibaly, N. G. Mounjouopou, E. Vaissaba, N. H. Luma, S. A. Mouelle, A. K. Njamnshi

**Affiliations:** ^1^Douala General Hospital, Faculty of Medicine and Pharmaceutical Sciences, The University of Douala, Douala, Cameroon; ^2^Douala General Hospital, Institut des Sciences de la Santé, Université des Montagnes, Douala, Cameroon; ^3^Douala General Hospital, Douala, Cameroon; ^4^Faculty of Medicine and Pharmaceutical Sciences, The University of Douala, Douala, Cameroon; ^5^Department of Internal Medicine in Douala General Hospital, Faculty of Medicine and Biomedical Sciences, The University of Yaoundé I, Douala, Cameroon; ^6^Department of Clinical Sciences, Faculty of Medicine and Pharmaceutical Sciences, The University of Douala, Douala, Cameroon; ^7^Department of Neurology, Yaoundé Central Hospital & Faculty of Medicine and Biomedical Sciences, University of Yaoundé I, Yaoundé, Cameroon

## Abstract

*Background.* The objective of this study was to describe complications in hospitalized patients for stroke and to determine the predictive factors of intrahospital mortality from stroke at the Douala General Hospital (DGH) in Cameroon. *Patients and Methods.* A prospective cross-sectional study was carried out from January 1, 2010 to December 31, 2012, at the DGH. All the patients who were aged more than 15 years with established diagnosis of stroke were included. A univariate analysis was done to look for factors associated with the risk of death, whilst the predictive factors of death were determined in a multivariate analysis following Cox regression model. *Results.* Of the 325 patients included patients, 68.1% were males and the mean age was 58.66 ± 13.6 years. Ischaemic stroke accounted for 52% of the cases. Sepsis was the leading complications present in 99 (30.12%) cases. Independent predicting factors of in-hospital mortality were Glasgow Coma Scale lower than 8 (HR = 2.17 95% CI 4.86–36.8; *P* = 0.0001), hyperglycaemia at admission (HR = 3.61 95% CI 1.38–9.44; *P* = 0.009), and hemorrhagic stroke (HR = 5.65 95% CI 1.77–18; *P* = 0.003). *Conclusion.* The clinician should systematically diagnose and treat infectious states and hyperglycaemia in stroke.

## 1. Introduction

Stroke is the leading cause of hospitalization in neurology in Sub-Saharan Africa [1, 2]. Adverse sequelae of stroke are common and mortality rate is high. Prognosis of stroke has been studied elsewhere including predictive factors of death. In Sub-Saharan Africa, stroke patients are generally hospitalized in the neurological service in Sub-Saharan Africa or in the intensive care unit since stroke units are not yet available. The aim of this study was to describe complications occurring in stroke patients and to determine the predictive factors of intrahospital mortality at the Douala General Hospital (DGH).

## 2. Patients and Methods

### 2.1. Study Setting

Douala, the economic capital of Cameroon, has a population of 3 million and an equatorial climate and is situated in the Gulf of Guinea. The DGH is a teaching hospital with 320 beds for paediatric, surgery, gynaecology and obstetrics, cobalt therapy, nephrology and haemodialysis, intensive care, emergency, and internal medicine departments. The imaging department uses a CT scan with 8 barettes and is functional 24 hours daily. Magnetic resonance imaging is available in Douala but only in the private sector (the cost of an MRI of the brain was 381.38€ in a country where the interprofessional minimal salary is 43.47€). Stroke patients are hospitalized in the neurology unit (NU) of the internal medicine department, and those with severe conditions at admission were hospitalized in the intensive care unit (ICU).

### 2.2. Study Design and Patient Management

A prospective longitudinal study was performed from January 1st. 2010 to December 31st. 2012. We recruited consecutively all the patients more than 15 years of age with clinical diagnosis of stroke [1] (confirmed by a CT scan). Patients with severe conditions like a Glasgow Coma Scale <8/15 or septic shock were directly admitted in the ICU while other cases were hospitalized in the NU. For each patient, sociodemographic, past medical history and clinical data were recorded. Time from onset to initial consultation was also accessed. The definitions of vascular risk factor are found in Table 1. At entrance, vital signs including blood pressure, pulse, respiratory rate, oxygen saturation, temperature, capillary glycemia, and urine analysis with a dipstick were recorded. Neurological assessment was done by a neurologist or intensive care specialist. Interpretation of CT scans was done by both radiologists and neurologists. Electrocardiography was systematically done for patient with ischemic strokes and for hypertensive patients with hemorrhagic strokes. For patients with ischemic stroke, transthoracic and supraaortic Doppler ultrasound was done, except for those with severe conditions. Blood samples were collected for a standard assessment including: a full blood count with platelet counts, urea and creatinine, electrolytes, fasting glucose, lipid profile, prothrombin time, cephaline-kaolin time, uric acid, C reactive protein, erythrocyte sedimentation rate, and HIV tests. Other tests were prescribed if required by the patient's conditions: chest X-ray, urine culture, hemoculture, and thick blood film to check for plasmodium falciparum. Followup was done daily for clinical evaluation, and complications were noted. In case of death, the staff had to be precise about the cause of death. Oxygen was administrated if ambient oxygen saturation was less than 94%. Paracetamol was used 1 g every six hours if temperatures superior to 37.5°C were noted. Prevention of deep venous thrombosis and stress ulcers was done using prophylactic dose of enoxaparin (40 mg) and omeprazole (20 mg), respectively. An insulin protocol was set up when capillary glycaemia was above 1.4 g/L. Concerning the blood pressure, nicardipine was given intravenously with electric syringe in case of high blood pressure with a target of 140 to 160 mmHg for systolic blood pressure in hemorrhagic stroke. In ischemic stroke, early elevated blood pressure was respected except when this was more than 220 mmHg for systolic pressure. Aspirin (100–250 mg per day) was given in ischemic stroke while curative dose of low weight molecular heparin (0.1 mL/kg twice daily) was used in case of atrial fibrillation with CHADS > 3, presence of intraluminal thrombus in a cerebral artery, or presence of blood clot in the heart. Antibiotics and artemether were used for bacterial infection and malaria, respectively. Thrombolysis is not yet effective in Cameroon.

### 2.3. Data Analysis

The SPSS software version 20 was used to analyse data. Chi square and Fisher tests were used to compare qualitative variables while the Student's *t*-test was performed for quantitative variables. *P* values <0.05 were considered statistically significant. To determine the predictive factors for death, univariate analysis was first done to look for an association between the variables and death in hospitalisation. Then the effect of the variables significantly associated with hospital mortality was studied in a multivariate analysis with the use of Cox model of proportional risk. All variables for which the *P* value was ≤0.02 in univariate analysis were included in the model. The demographic variables were sex (i.e., male or female) and age divided into 4 groups: <45 years, 45 to 54 years, 55 to 64 years, and >64 years. The first age group served as the reference for analysis. The time interval between the onset of symptoms and the first consultation, then consultation at the DGH, was dichotomised into *⩽*6 hours and >6 hours. The known comorbidities at admission notably hypertension, diabetes, dyslipidaemia, alcohol consumption, tobacco consumption, HIV infection, and past medical history of stroke were included in the analysis. The clinical variables were the Glasgow Coma Score >8 or *⩽*8/15 and temperature > or <37.5°C. The capillary blood sugar on admission (≤1.4 and >1.4 g/L), the serum creatinine level (≤13 and >13 mg/L), uraemia (≤0.45 and >0.45 g/L), CRP (<6 mg/L, 6 to 24 mg/L and >24 mg/L), and uricaemia (≤70 mg/L and >70 mg/L) were the biological variables. The nature of the stroke (ischaemia or haemorrhage) was considered as another variable. The last variable was the existence or absence of complications during the hospitalization period.

### 2.4. Ethical Considerations

We obtained clearance from the DGH Ethics Committee. The identity of patient was strictly confidential. No specific consent was signed by patients.

## 3. Results

A total of 325 patients were recruited with 258 (79.39%) in the NU and 67 (20.61%) in the ICU. Males represented 201 (68.1%) of case with a male to female ratio of 1.62.  Table 2 presents the characteristics of the study population. 127 (39.1%) came directly from their home while the remaining had been referred by other health centers. The mean delay from the onset of stroke and the initial consultation was 47.36 ± 18.48 hours (1 to 441.75 hours). The mean delay to consult in the DGH was 96.37 ± 64.99 hours (1 to 720 hours).

Table 3 shows the clinical parameters on admission. The Glasgow coma score was inferior or equal to 8/15 in 55 patients (16.3%). Urine analysis showed a suspicion of urinary tract infection based on the presence of both nitrite and leucocytes on 34 patients (10.46%) and confirmed by positive culture. Ischemic and haemorrhagic strokes represent, respectively, 52% and 48%.  Table 4 shows complications during admission with infectious condition as the leading complication with 37.53% of cases. The global (NU and ICU) mean duration of hospitalization was 8.56 ± 6.35 days. In the ICU, the mean duration of hospitalization was 4.38 ± 4.32 days and 9.67 ± 6.36 days in the NU (*P* < 0,001). During this hospitalization period, the global in-hospital mortality rate was 26. 8%. In the NU, the mortality rate was 13.95% (36/258) while 76.12% (51/67) in the ICU.

After univariate analysis, only male, dyslipidemia, previous stroke, Glasgow score scale inferior to 8/15, temperature superior to 37.5°C, hyperglycaemia, elevated urea and seric creatinine, and lastly the hemorrhagic stroke were associated with in-hospital mortality; while alcohol consumption and tobacco appeared to be protective. After multivariate analysis following Cox regression model, only the GCS < 8/15 (OR = 13.41, CI 4.86–36.9; *P* = 0.0001), hyperglycaemia (OR = 3.63, CI 1.38–9.44; *P* = 0.009), and the haemorrhagic nature of the stroke (OR = 5.65, CI 1.77–18.02; *P* = 0.003) appeared as independent predictive factors 5 of mortality (Table 5).

## 4. Discussion

The aim of the study was to describe the complications of stroke and determine the predictive factors of in-hospital mortality. For a mean hospital stay of 8.58 ± 6.35 days, the global hospital mortality was 26.8%. This hospital mortality is close to that reported by Touré et al. [3] in Senegal. They also recruited patients in the neurology and intensive care units. Other Sub-Saharan authors describe that mortality rates 9.5 and 15.5% in patients followed up solely in neurology [4, 5]. Western studies that included both ischaemic and haemorrhagic strokes report hospital mortality rates of between 4.8% [6] and 16% [7]. A multicentric Asiatic study similar to the current study reported 12.5% [8]. These differences could be linked to the quality of the global health system: systematic screening of cardiovascular conditions, the delays in management, and most of all the universal access to healthcare. In Sub-Saharan Africa, it is often after a stroke that a patient is discovered to have hypertension, diabetes, and dyslipidemia often with involvement of several target organs. There are no social security schemes yet for illness and the patient's healthcare needs are paid for by the patients or their family according to their financial stamina. Fifty-one patients (15.69%) had at least one complication on admission, made up mainly of infections first of which were bronchopulmonary and urinary infections. This predominant place of pulmonary and urinary infectious complications had been reported by Langhorne et al. [7]. In our context, to eat well while ill is a sign that assures the family and so before even reaching the neurology wards, many patients already have inhalation pneumonitis due to food ingestion. Urinary tract infections were more frequent in diabetic patients and were often asymptomatic explaining the need for systematic screening using simple urine dipstick tests in our daily practice. The main causes of death were septic states that often lead to multivisceral failure. Preventing and fighting vigorously against infections in our milieu could reduce their impact on hospital mortality. Early epileptic seizures were found in 11.07% of the patients. Hospital-based African studies do not mention these seizures [3–5] whereas Langhorne et al. reported a seizure rate of 3% [7]. This difference has got no explanation apart from the fact that most epileptic seizures occur mostly in cerebral haemorrhage which represents almost 48% in our sample as against only 11% in this British study. The delay in management did not influence mortality significantly despite the fact that most patients consulted 96.37 ± 64.99 hours (about 4.01 days) after the onset of stroke symptoms, a delay similar (4 days) to that in the British study [7].

We searched globally (without distinction between cerebral ischaemia and haemorrhage) since in our context cerebral imaging is not often available; it is therefore important for us to scan through the factors that can influence mortality. Alcohol and tobacco consumption appeared to have a protective action on mortality risk without us having an explanation for such a paradox. Only coma, hyperglycaemia on admission, and the haemorrhagic nature of the stroke were independent predictive factors of hospital mortality. In Sub-Saharan Africa, only a study in Senegal looked for prediction, and only coma and a past history of stroke were independent predictive factors of the risk of hospital mortality [3]. Western and Asiatic studies have looked at the predictive factors with respect to the ischaemic or haemorrhagic nature of the stroke. For cerebral ischaemia, disturbances of alertness, cardiac arrhythmia, left ventricular hypertrophy, the severity of the neurological deficit are commonly reported factors [6, 8, 9]. Age is not described by all authors. Radiological abnormalities were cited as predictive factors of hospital mortality notably: the existence of early signs of cerebral ischaemia on the CT scan, the presence of an intraluminal thrombus, or proximal arterial obstruction [10]. Concerning cerebral haemorrhage, alteration of consciousness and weakness of a limb were reported by Baptista et al. [6] as predictive factors of hospital mortality. Asiatic studies described only diabetes mellitus as a predictive factor of in-hospital mortality [8].

## 5. Conclusion

Hospital mortality of stroke is particularly high in Douala. The main complications of stroke are due to septic conditions. The medical practitioners need to prevent and vigorously combat infections and hyperglycaemia occurring during stroke. Comatose patients and/or those with cerebral haemorrhage have to benefit from improved and better surveillance. Implementation of stroke units will be a necessity to reduce the impact of stroke mortality.

## Figures and Tables

**Table 1:** Definition of vascular risk factors.

Vascular risk factor	Definition
Hypertension	Patient with medical history of hypertension, treated or not.
Diabetes mellitus	Patient with medical history of diabetes, treated or not.
Dyslipidemia	Patient with medical history of dyslipidemia, treated or not with one of these conditions
Sleep apnoea disease	Suspected in patient with 3 of these conditions: (i) Snoring when sleeping (ii) Apnea during sleeping (iii) Excessive diurnal sleeping (iv) Can be associated with obesity
Alcohol consumption	Daily alcohol intake >60 g/L
Obesity	2 methods were used to determine overweight or obesity: (i) If the body mass index is >30: obesity, BMI between 25 and 30 is overweight (ii) When it is impossible to have the BMI, we used the abdominal circumference >102 cm in male and >88 cm in female

**Table 2:** Characteristic of patients.

Characteristic of patients	Number	Percentage (%)
Sex (male/female)	201/124	68.1/38.2
Age	58.66 ± 13.06	—
Known vascular risk factors at admission:		
(i) High blood pressure	227	69.84
(ii) Alcohol consumption	92	28.3
(iii) Diabetes mellitus	67	20.61
(iv) Overweight/obesity	59	18.15
(v) Tobacco	52	16.0
(vi) Previous stroke	38	11.69
(vii) Cardioembolic disease	36	11.07
(viii) Dyslipidemia	29	8.9
(ix) HIV infection	10	3.07

**Table 3:** Clinical parameters of patients at admission.

	Mean	Standard deviation	Minimum	Maximum
Systolic BP (mmHg)	168.38	33.60	88	255
Diastolic BP (mmHg)	100.88	20.50	50	171
Pulse (/min)	84.41	19.32	26	169
Respiratory rate (/min)	23.75	9.24	12	99
Temperature (°C)	37.29	0.76	35.8	40.30
Capillary glycaemia (g/L)	1.47	0.77	0.23	5.99
Glasgow, scale (/15)	12.25	3.35	3	15

BP: blood pressure.

**Table 4:** Complications on admission and those occurring during hospitalisation.

Complications	At entrance	During admission	Total
Bronchopneumonia	10 (3.07)	40 (12.3)	50 (15.06)
Urinary tract infection	34 (10.46)	15 (4.6)	49 (15.06)
Seizures	32 (9.84)	4 (1.23)	36 (11.07)
Deglutition disturbance	16 (4.92)	6 (1.84)	22 (6.77)
Phlebitis	0	19 (5.84)	19 (5.84)
Intracranial hypertension	9 (2.76)	9 (2.76)	18 (5.52)
Bedsore	4 (1.23)	9 (2.76)	13 (3.99)
Renal failure	4 (1.23)	6 (1.84)	10 (3.07)
Severe anaemia	0	6 (1.84)	6 (1.84)
Malaria	2 (0.61)	3 (0.92)	5 (1.53)
GIB	0	4 (1.23)	4 (1.23)
Gout	0	4 (1.23)	4 (1.23)
Deep venous thrombosis	3 (0.92)	0	3 (0.92)

GIB: gastrointestinal bleeding.

**Table 5:** Death predicting factors of in-hospital mortality in patients with stroke.

	Survival	Death	Univariate analysis		Multivariate analysis	
	*N* (%)	*N* (%)	OR (95% CI)	*P*	OR (95% CI)	*P*
Sex						
Female	85 (68.5)	39 (31.5)	0.75 (0.49–1.14)	0.18	0.72 (0.23–2.19)	0.56
Male	153 (76.1)	48 (23.9)				
Age (years)						
<45	31 (68.9)	14 (31.1)		
45–54	58 (77.3)	17 (22.7)	0.68 (0.33–1.42)	0.32
55–64	71 (71)	29 (29)	0.85 (0.45–1.61)	0.62		
≥65	78 (74.3)	27 (25.7)	0.80 (0.42–1.52)	0.51		
Time 1*						
≤6 hours	147 (71)	60 (29)	0.77 (0.49–1.21)	0.27		
>6 hours	91 (77.1)	27 (22.9)				
Time 2**						
≤6 hours	66 (75.9)	21 (24.1)	1.13 (0.69–1.85)	0.63		
>6 hours	172 (72.3)	66 (27.7)				
Comorbidities***						
HBP	167 (73.6)	60 (26.4)	0.91 (0.58–1.43)	0.68		
Diabetes	50 (74.6)	17 (25.4)	0.94 (0.56–1.60)	0.83		
Dyslipidemia	26 (89.7)	3 (10.3)	0.35 (0.11–1.12)	0.08	0.5 (0.06–4.13)	0.52
Stroke^§^	31 (81.6)	7 (18.4)	0.62 (0.29–1.34)	0.20	0.46 (0.09–2.16)	0.33
Alcohol	80 (87)	12 (13)	0.34 (0.18–0.62)	0.0001	0.43 (0.12–1.58)	0.21
Tobacco	48 (92.3)	4 (7.7)	0.23 (0.08–0.62)	0.004	1.02 (0.16–6.52)	0.99
HIV	8 (80)	2 (20)	0.67 (0.16–2.71)	0.57		
Glasgow, (/15)						
≤8	10 (18.2)	45 (81.8)	9.34 (6.08–14.33)	0.0001	13.41 (4.86–36.9)	0.0001
>8	228 (84.4)	42 (15.6)				
Temperature (°C)						
≤37.5	196 (80.3)	48 (19.7)	2.78 (1.82–4.25)	0.0001	2.17 (0.76–6.23)	0.15
>37.5	42 (51.9)	39 (48.1)				
Glycemia (g/L) capillary						
≤1.4	150 (79.4)	39 (20.6)	1.87 (1.19–2.92)	0.007	3.61 (1.38–9.44)	0.009
>1.4	68 (64.8)	37 (35.2)				
Serum creatinine (mg/L)						
≤13	152 (80.9)	36 (19.1)	1.74 (1.08–2.80)	0.02	0.75 (0.19–3.02)	0.69
>13	66 (67.3)	32 (32.7)				
Urea (g/L)						
≤0.45	152 (81.3)	36 (18.7)	1.79 (1.07–2.98)	0.02	1.57 (0.41–6.08)	0.51
>0.45	52 (66.7)	26 (33.3)				
CRP (mg/L)						
<6	77 (92.8)	6 (7.2)				
6–24	39 (83)	8 (17)	1.79 (0.62–5.19)	0.29	1.03 (0.24–4.36)	0.97
>24	35 (71.4)	14 (28.6)	2.67 (1.01–7.08)	0.048	0.89 (0.23–3.4)	0.87
Uric acid (mg/L)						
≤70	35 (87.5)	5 (12.5)	1.68 (0.49–5.80)	0.41
>70	59 (92.2)	5 (7.8)		
Complications during admission	70 (66.7)	35 (33.3)	1.06 (0.62–1.81)	0.41		
Stroke's type						
Ischemic	148 (87.1)	22 (12.9)	3.32 (2.05–5.40)	0.0001	5.65 (1.77–18.02)	0.003
Hemorrhagic	90 (58.1)	65 (41.9)				

Time 1: delay from stroke onset and the first consultation; Time 2: delay from stroke onset and consultation at Douala General Hospital.

Comorbidities: known vascular risk factors during admission; HBP: high blood pressure; ^§^past medical history of stroke.
